# The Cooperative Functions of the EBNA3 Proteins Are Central to EBV Persistence and Latency

**DOI:** 10.3390/pathogens7010031

**Published:** 2018-03-17

**Authors:** Christine T. Styles, Kostas Paschos, Robert E. White, Paul J. Farrell

**Affiliations:** Molecular Virology, Department of Medicine, Imperial College London, London W2 1PG, UK; christine.styles10@imperial.ac.uk (C.T.S.); k.paschos@imperial.ac.uk (K.P.); robert.e.white@imperial.ac.uk (R.E.W.)

**Keywords:** Epstein–Barr virus, EBNA3 proteins, epigenetic regulation, viral oncogenes, viral tumour suppressor, CDKI regulation

## Abstract

The Epstein–Barr nuclear antigen 3 (EBNA3) family of proteins, comprising EBNA3A, EBNA3B, and EBNA3C, play pivotal roles in the asymptomatic persistence and life-long latency of Epstein–Barr virus (EBV) in the worldwide human population. EBNA3-mediated transcriptional reprogramming of numerous host cell genes promotes in vitro B cell transformation and EBV persistence in vivo. Despite structural and sequence similarities, and evidence of substantial cooperative activity between the EBNA3 proteins, they perform quite different, often opposing functions. Both EBNA3A and EBNA3C are involved in the repression of important tumour suppressive pathways and are considered oncogenic. In contrast, EBNA3B exhibits tumour suppressive functions. This review focuses on how the EBNA3 proteins achieve the delicate balance required to support EBV persistence and latency, with emphasis on the contribution of the Allday laboratory to the field of EBNA3 biology.

## 1. Epstein–Barr Virus

The lymphotropic gamma-herpesvirus Epstein–Barr virus (EBV) asymptomatically and persistently infects the majority of the worldwide human population. Despite being a ubiquitous virus, it is one of the most transforming viruses identified, and is aetiologically associated with a variety of B cell malignancies, including Burkitt’s lymphoma (BL), Hodgkin’s lymphoma (HL), and diffuse large B cell lymphoma (DLBCL), alongside post-transplant lymphoproliferative disease, nasopharyngeal carcinomas, and some gastric carcinomas [1]. Upon infection of mature B lymphocytes in vitro, EBV efficiently induces transformation of B cells into continuously proliferating lymphoblastoid cell lines (LCLs). These LCLs express nine latency associated viral proteins; there are six nuclear antigens (EBNA1, EBNA2, EBNA3A, EBNA3B, EBNA3C, and EBNA-LP) and three latent membrane proteins (LMP1, LMP2A, and LMP2B), alongside several RNA species [2]. In vivo, the biology of EBV is intimately linked to that of B cell differentiation. It is thought that EBV infection drives naïve B cells to proliferate and differentiate into activated B-blasts, which phenotypically resemble LCLs. These activated B-blasts then migrate through germinal centres where they further differentiate, resulting in resting memory B cells that carry the EBV genome as extra-chromosomal episomes, forming long-lived reservoirs of EBV infection [3]. During transition through the germinal centre there is a sequential silencing of viral gene expression, so that no viral proteins are expressed in resting memory B cells [2].

## 2. The EBNA3 Family

**Figure 1:**
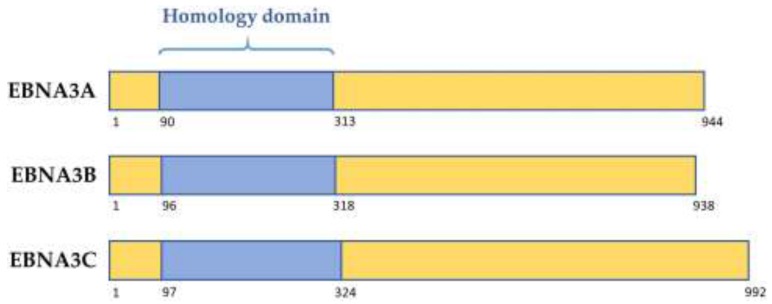
Schematic showing structure of the homology domain between the Epstein–Barr virus (EBV) EBNA3 family of proteins. Numbers denote amino acid position within the prototype strain B95.8 protein.

The latency-associated EBNA3 family of proteins comprises 3 large proteins called EBNA3A, EBNA3B, and EBNA3C, each composed of over 900 amino acids. These non-redundant proteins are thought to have arisen from tandem duplication events during the evolution of old world lymphocryptoviruses. Their genes are structurally similar, with each consisting of a short 5′ coding exon and long 3′ coding exon, and share limited but significant sequence homology. Each of the EBNA3 proteins features a proline rich region, as well as a “homology domain” of approximately 220–230 amino acids near the N-terminus, in which there is 20–30% identity between the EBNA3s (Figure 1, [4,5]). The EBNA3s are predicted to share similar secondary structures, despite having only modest homology overall [4]. EBNA3 mRNA transcripts are initiated from the Cp latency promoter producing large, alternatively spliced mRNAs that are only expressed in B cells. Production of EBNA3 proteins is thought to be tightly regulated, and the proteins are very stable, as protein levels and turnover are low [6,7]. 

EBV recombinant viruses containing various EBNA3 genetic modifications have been generated using bacterial artificial chromosome (BAC) technology, allowing detailed study of the EBNA3 proteins in the context of infection, B cell transformation and maintenance of viral latency. EBNA3 knockouts (KO) have been generated using mutations and deletions [8,9], alongside conditional systems. These conditional systems use an oestrogen receptor fused to the open reading frames of EBNA3A and/or EBNA3C, making functional expression dependent on the activating ligand 4-hydroxytamoxifen (HT). The HT stabilises the fusion protein, allowing nuclear localisation and functional expression [10,11,12,13,14]. Epitope tagged EBNA3 fusion genes in EBV BACs have been used in chromatin immunoprecipitation sequencing (ChIP-seq) experiments to identify thousands of EBNA3 binding sites across the genome [15,16,17,18]. 

EBNA3 proteins are well characterised regulators of transcription. Exon-microarray analysis using the EBV-negative Burkitt’s lymphoma (BL) cell line BL31 infected with mutant EBNA3 viruses revealed that the EBNA3 proteins transcriptionally regulate over 1000 host cell genes. A substantial number of these genes show cooperative regulation between multiple EBNA3 proteins. Most of this co-regulation involves EBNA3C working in cooperation with either EBNA3A or EBNA3B or both, with only modest cooperation between EBNA3A and EBNA3B in the absence of EBNA3C. Moreover, deletion of EBNA3C had the most significant impact on host gene regulation, with gene repression dominating differential host gene expression [8]. ChIP analysis demonstrated that EBNA3A and EBNA3C have significant colocalisation across the genome, as do EBNA3B and EBNA3C. However, colocalisation of EBNA3A and EBNA3B in the absence of EBNA3C was extremely rare [18]. EBNA3 proteins do not bind to DNA directly, instead, transcriptional regulation is achieved through interactions with cellular DNA binding factors (see Section 3). 

Both EBNA3A and EBNA3C are considered oncogenic. In an early demonstration of this, EBNA3C was shown to cooperate with oncogenic (Ha-)Ras during transformation of primary rat embryo fibroblasts [19]. The oncogenic functions of EBNA3A and EBNA3C differ from the seemingly antagonistic EBNA3B, despite clear cooperative regulation of a variety of genes [20]. Alongside EBNA1, EBNA2, EBNA-LP, and LMP1, both EBNA3A and EBNA3C are required for the efficient transformation of B cells [21,22,23,24,25]. EBNA3C is essential for B cell transformation, and EBNA3A is important, with EBNA3A-null cell lines exhibiting reduced proliferative capacity [10,12,14,26]. In contrast to the necessity of EBNA3A and EBNA3C for efficient B cell immortalisation, EBNA3B is completely dispensable for the transformation of B cells in vitro, and appears to act as a tumour suppressor in vivo ([27], see Section 6).

## 3. EBNA3 Regulatory Mechanisms

### 3.1. Recruitment

**Figure 2:**
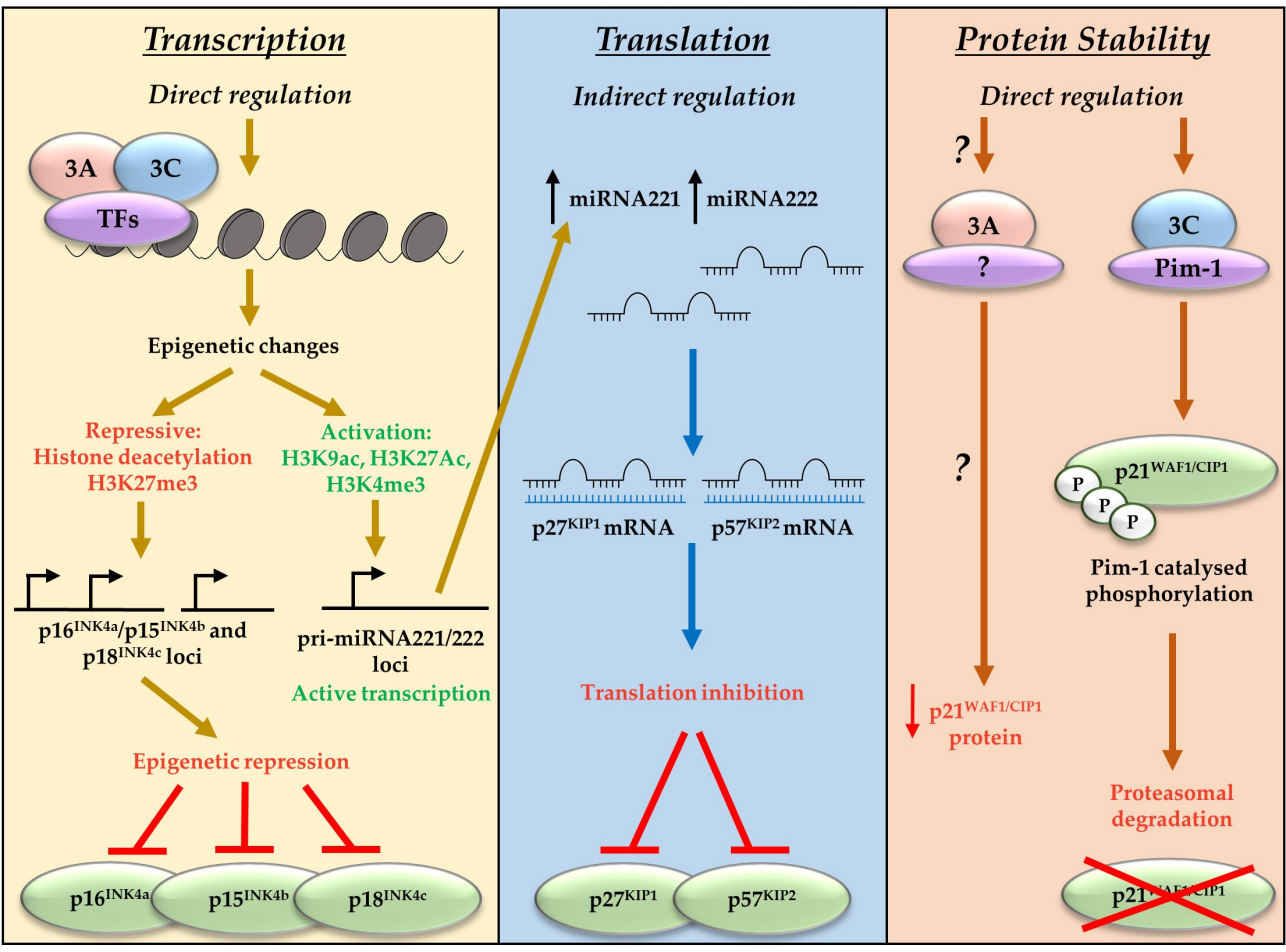
EBNA3A and EBNA3C regulate CDKI at a transcriptional, translational, and protein levels. EBNA3A and EBNA3C bind cell transcription factors (TFs) including RBP-Jκ, CBF, and IRF4. This results in polycomb complex recruitment and deposition of chromatin marks on the loci of target genes. These chromatin marks can either repress transcription (the CDKIs p16^INK4a^, p15^INK4b^, and p18^INK4c^) or activate transcription (miRNA221/222). EBNA3-mediated activation of miRNA221/222 leads to subsequent repression of the CDKI p27^KIP1^ and p57^KIP2^ via translation inhibition. EBNA3A and EBNA3C can also repress CKDI by targeting them for degradation. EBNA3A mechanisms are unclear, but EBNA3C has been shown to mediate Pim-1 catalysed phosphorylation and subsequent proteasomal degradation of p21^WAF1/CIP1^.

Although the EBNA3 proteins regulate many target genes and robustly associate with chromatin, they do not bind DNA directly. Instead, the EBNA3 proteins bind many cell transcription factors to facilitate viral regulation of host genes. Of these factors, the Notch signalling pathway DNA-binding factor RBP-Jκ (also called CBF1) was the first to be identified as a binding partner for all EBNA3s [28,29], with the interaction mapped to the EBNA3 homology domain (See Figure 1, [2,28,30,31]). RBP-Jκ is also bound by the EBV transactivator EBNA2 in a mutually exclusive manner to the EBNA3s, suggesting some interplay of functions [32]. In transient reporter assays, each EBNA3 was able to inhibit activation of viral promoters LMP2A and Cp by EBNA2, indicating the importance of RBP-Jk in EBNA3 function, and showing EBNA3s to be transcriptional regulators [5,29,33,34]. This led to the hypothesis that RBP-Jκ is the recruiter of the EBNA3s to chromatin. The importance of RBP-Jk was further corroborated when it was shown that interaction of RBP-Jκ with EBNA3A and EBNA3C is required to achieve the genetic reprogramming of B cells that allow continuous proliferation of LCLs [35,36,37]. The mechanism for this is still unclear. 

RBP-Jκ is, however, absent from many known EBNA3 binding sites as revealed by ChIP-seq experiments exploring EBNA3 genome-wide localization [16,17,18,38,39]. In addition, at sites where RBP-Jk was present, it was shown to be dynamically recruited to chromatin concurrently with EBNA3C [30]. The same studies also revealed that the EBNA3s bind at sites containing binding motifs for other transcription factors, or sites where numerous transcription factors are known to bind (e.g., PU.1, EBF1, RUNX3, BATF, IRF4), potentially recruiting the EBNA3s there. IRF4 has been shown to be important for EBNA3C recruitment on to chromatin, by assessing localization of exogenously expressed EBNA3A, EBNA3B, or EBNA3C in cells either lacking or expressing IRF4 [39]. In a separate study, it was shown that EBNA3C and EBNA3B (and to a lesser extent EBNA3A) all bind to core binding factor β (CBFβ), which heterodimerizes with the transcription factor RUNX3 to form the core binding factor (CBF). EBNA3 binding to target gene loci was reduced when CBF was depleted by shRNA against CBFβ, indicating its importance for EBNA3 recruitment to chromatin [18]. So far there is direct evidence for only these two transcription factors (IRF4 and CBF), mediating recruitment of EBNA3B and/or EBNA3C. It is unknown whether they are the only ones, and unclear whether they act independently at different sites, or cooperatively at sites, where both bind together with EBNA3C. IRF4, and to a lesser extent EBF1, were also recently shown to be important in recruitment of EBNA3A to the EBNA3A-regulated locus *STK39* [40]. It is currently unclear whether this effect is independent of EBNA3C.

### 3.2. Changes in Chromatin Architecture

All EBNA3s localise to chromatin with histone marks characteristic of enhancer elements [16,17,18,38,39], indicating that long distance chromatin interactions are a prominent feature of gene regulation by EBNA3. EBNA3 binding sites found distal to promoters of regulated genes (*CXCL10/CXCL9* [41], *ADAM28/ADAMDEC1* [38]) further corroborated this, and EBNA3-mediated chromatin looping was eventually directly shown by chromosome conformation capture experiments for EBNA3A and EBNA3C (reviewed in [32]). Specifically, EBNA3C, and in part, EBNA3A, are thought to facilitate repression of the *ADAM28-ADAMDEC1* locus by promoting a long-range interaction between an intergenic enhancer and their respective promoters [30,38]. Additionally, EBNA3C promotes chromosome looping between the downstream enhancer and promoter of *WEE1,* leading to gene repression [38]. In contrast, *BCL2L11* (BIM) and *CTBP2* repression are thought to result from EBNA3-mediated disruption of enhancer–promoter interactions [38,42]. Furthermore, EBNA3A and EBNA3C can also activate transcription by promoting chromosome looping between distal enhancer elements and the transcription start site of the pri-miRNA of the miRNA 221/222 cluster [12]. Together, these observations suggest that EBNA3C and probably EBNA3A can both engage and disrupt long range chromatin interactions, and that these structural changes can either enhance or repress gene transcription, probably in ways that can only be ascertained on a gene-by-gene basis.

### 3.3. Repressors and Activators

The ability of the EBNA3s to mediate both host gene activation and repression was clear from a series of microarray experiments that interrogated the transcriptional profile of cells with or without expression of each EBNA3 [8,26,43]. This is despite results from transient reporter assays mentioned above that suggested all EBNA3s are repressors. For EBNA3A/EBNA3C [12] and EBNA3C [30], direct transcriptional activation of host genes has been shown, but not for EBNA3B. Not surprisingly, EBNA3A and EBNA3C binding sites have been identified within ”contact domains” (genomic regions shown to come into contact through looping) of both up- and downregulated target genes. In contrast, EBNA3B binds significantly only within contact domains of genes identified as downregulated by EBNA3B, suggesting that EBNA3B functions directly only as a repressor [18].

How the different outcomes of repression or activation are determined for EBNA3A and EBNA3C is unclear. One could envisage that the EBNA3 proteins act always in the same way, and the chromatin context of each gene determines the outcome, but this has not yet been shown. It seems that the outcome of looping (i.e., facilitation or disruption of looping) is not the only determinant. There can be facilitation of looping and two different outcomes, for example, miRNA221/222 with activation [12] and *ADAM28/ADAMDEC1* with repression [38]; in both these cases, EBNA3A and EBNA3C regulate. There can also be repression by either facilitation or disruption of looping, notably at *ADAM28/ADAMDEC1* with facilitation, and at *BCL2L11* with disruption [38], under EBNA3A/EBNA3C regulation.

### 3.4. EBNA3 Interactors That Facilitate Regulation

Regulatory mechanisms of EBNA3C are much better characterised than those of EBNA3A, and less is known about EBNA3B, with the current lack of an EBNA3B-ERT2 conditional virus limiting investigation. Part of their action is explained by their antagonism with the viral transactivator EBNA2 for binding to RBP-Jκ. This, however, is not the whole story, because of EBNA3 binding elsewhere and EBNA3A and EBNA3C also acting as activators themselves. Several transcription cofactors that could facilitate regulation have been found to interact with EBNA3A and EBNA3C. These include the histone deacetylases (HDAC1 and HDAC2) and the co-repressor CtBP for EBNA3A, and HDAC1/2, CtBP, SIN3 transcription regulator family member A (SIN3A), nuclear receptor co-repressor (NCoR) and histone acetyltransferase p300 for EBNA3C [44,45,46,47,48]. Of these, only CtBP has been confirmed to be directly involved in EBNA3-mediated gene repression by genetic analysis using recombinant EBV viruses expressing mutant EBNA3A and/or EBNA3C that cannot bind to CtBP. Here, the requirement for CtBP interaction was demonstrated for EBNA3-mediated repression of p16^INK4a^ in B cells, but the mechanistic basis of this requirement remains uncertain [10]. 

The p300 histone acetyltransferase is a co-activator, and its association with EBNA3C might explain EBNA3C’s ability to activate. Enrichment of p300 at EBNA3C-activated gene AICDA was found in response to EBNA3C presence [49], but more EBNA3 activated genes will have to be studied to clarify whether additional undiscovered factors may also contribute.

HDACs, NCoR, and SIN3A are generally thought of as repressors, and are all associated with histone deacetylation—a primary epigenetic mark associated with repression. In all cases of EBNA3-mediated repression investigated on a gene-by-gene basis, histone deacetylation has been observed. Importantly, when the process of histone deacetylation was tracked together with establishment of repression, they were found to be concurrent [30,41].

EBNA3-mediated gene silencing through association with transcription factors often involves localising polycomb repressive complexes 1 and 2 (PRC1, PRC2) to target genes. Interestingly, PRC1 component BMI1 was found to be recruited to genes during establishment of repression, with kinetics that matched those of histone deacetylation, suggesting that it might be important at the initial stage [30]. BMI1 knockdown has also been shown to lead to de-repression of the EBNA3A/3C-repressed gene BIM [15], although it should be noted that PRC1 has also been implicated in gene activation [50]. It remains to be shown if this is relevant in the EBNA3 context.

In contrast to PRC1, the involvement of PRC2 in repression of EBNA3 target genes is well established, and can often result in deposition of the repressive histone mark H3K27me3, catalysed by the methyltransferase subunit (EZH2) of PRC2 [10,13,15,30,40,41,51,52]. However, ChIP-seq analyses have shown that H3K27me3 colocalises with very few EBNA3 binding sites. In addition, H3K27me3 deposition was found to lag behind establishment of repression, suggesting that this epigenetic mark is a consequence, rather than the driver of initial repression in these cases [30,41]. This lag might also explain why for most EBNA3-regulated genes the timeframe of regulation is several days, with differently timed waves of epigenetic marks that have an additive effect. The additive effect of H3K27me3 in repression has been suggested by knockdown of PRC2 component SUZ12 or treatment with an inhibitor of EZH2 (the catalytic subunit of PRC2)—both causing de-repression [15,42]. Importantly, a recent study of the STK39 gene (the only gene described in detail so far that is solely repressed by EBNA3A) showed that PRC2 and H3K27me3 are important for the initial establishment of repression [40].

The apparent complexity of EBNA3 mechanisms of action is probably the result of the many transcription factors they seem to interact with. Regardless, general principles have emerged. Their association with chromatin is usually mediated by more than one DNA-binding transcription factor, probably in a locus-specific manner. They are mostly recruited to enhancer elements, distal to the promoters they regulate, which means that changes in chromatin architecture are necessary for their action. This can be either by enhancing or disrupting chromatin loop formation. There is evidence for direct activation only by EBNA3A and EBNA3C, whereas EBNA3B seems to act only as a repressor. The regulation they confer is epigenetic, primarily through changes in histone acetylation, which in some cases lead to changes in histone methylation with additive effects on repression.

## 4. EBNA3A and EBNA3C Regulate Anti-Proliferative and Tumour Suppressive Pathways

A complex system of phosphorylation and feedback loops regulates cell division to prevent aberrant proliferation and cancer progression. EBNA3A and EBNA3C have been shown to circumvent these pathways by interfering with intrinsic cell cycle associated factors that prevent anti-proliferation and suicidal response pathways. The best characterised is EBNA3A- and EBNA3C-mediated repression of various cyclin-dependent kinase inhibitors (CDKIs) [20]. Deregulation of the complex and tightly coordinated molecular machinery that controls the cell cycle, mediated through the concerted activity of EBNA3A and EBNA3C, likely plays a vital role in efficient EBV-mediated B cell transformation and subsequent viral persistence [43,53].

### 4.1. Direct Epigenetic Repression of the Tumour Suppressor and CDKI p16^INK4a^

EBV-mediated repression of the CDKI and tumour suppressor p16^INK4a^ was first shown using a recombinant virus containing EBNA3C fused to oestrogen receptor. This showed that functional EBNA3C is essential to restrain expression of p16^INK4a^ [11]. Inactivation of EBNA3C in conditional LCLs leads to progressively increased levels of p16^INK4a^ mRNA and protein, with correspondingly reduced levels of hyperphosphorylated forms of the retinoblastoma protein (Rb), a cessation of proliferation, and more cells in the G1 phase of the cell cycle [10,11,43]. Increased p16^INK4a^ levels were ablated by transfection of a plasmid expressing EBNA3C and by stabilisation of the conditional EBNA3C [11]. The *CDKN2A* gene encodes p16^INK4a^, and also (by an alternative reading frame) encodes p14^ARF^, which is also repressed by EBNA3C [54]. However, the consequences are functionally distinct. The p16^INK4a^ protein interacts with the cyclin dependent kinases CDK4 and CDK6, preventing phosphorylation of the retinoblastoma (Rb) protein leading to cell cycle arrest, whereas p14^ARF^ interacts with MDM2, resulting in p53 activation and subsequent cell cycle arrest or apoptosis [55]. Analysis of EBNA3A knockout and conditional LCLs has demonstrated that EBNA3A also plays a role in repressing both p16^INK4a^ and p14^ARF^ [10,26,54]. There is a cumulative effect on p16^INK4a^ when both EBNA3A and EBNA3C are inactivated, with elevated levels of p16^INK4a^ mRNA compared to when EBNA3A or EBNA3C are inactivated independently [13]. The importance of p16^INK4a^ repression during B cell transformation by EBV was highlighted by evidence that EBNA3C becomes unnecessary for transformation and sustained proliferation when p16^INK4a^ is functionally inactive. In an elegant experiment, Skalska et al. showed that EBNA3C-null LCLs can be readily produced from B cells derived from an individual with a homozygous deletion in *CDKN2A* that inhibits all known p16^INK4a^ functions. This demonstrated that EBNA3C-mediated repression of p16^INK4a^ plays a crucial role in the transformation of B cells by EBV [43].

ChIP analysis indicates EBNA3A and EBNA3C directly repress p16^INK4a^ through epigenetic chromatin modification, via deposition of the polycomb-associated repressive mark H3K27me3, and reduction of the activation mark H3K4me3 at the *CDKN2A* locus [10,54]. Moreover, epigenetic regulation of *CDKN2A* by EBNA3s is maintained in the absence of functional p16^INK4a^, further indicating *CDKN2A* is a direct target of EBV [43]. The chromatin remodelling of *CDKN2A* by EBNA3A and EBNA3C was shown to require interaction with the transcriptional co-repressor CtBP [10]. CtBP is the collective term for the proteins CtBP1 and CtBP2, which promote cell survival and are associated with repression of various tumour suppressors (as reviewed in [56]). EBNA3A and EBNA3C, but not EBNA3B, have been shown to interact with CtBP, and they are readily immunoprecipitated together from LCL extracts [44,47]. Mutating the CtBP binding sites within EBNA3A and EBNA3C compromises transformation of primary B cells infected with recombinant EBV, and similarly compromises transformation of rodent embryo fibroblasts following transfection with EBNA3A or EBNA3C [10,44,47]. In B cells, mutation of CtBP binding sites within EBNA3A and EBNA3C correlates with significantly reduced H3K27me3 at the *CDKN2A* locus, alongside increased H3K4me3. The resulting increases in p16^INK4a^ mRNA and protein are thought to contribute to the impaired outgrowth of primary B cells infected with EBV containing mutated CtBP binding sites in EBNA3A and EBNA3C, and the reduced proliferative capacity of resulting LCLs [10].

Alongside *CDKN2A*, EBNA3 proteins have also been shown to repress the adjacent gene *CDKN2B.* This gene encodes the related INK4 family protein p15^INK4b^, which performs similar functions to p16^INK4a^ [55]. EBNA3A-mediated transcriptional repression was shown to occur via interactions with the transcriptional factor MIZ1, leading to subsequent deposition of the repression histone mark H3K27me3 in close proximity to the transcription start site, as seen with p16^INK4a^ repression [43,57]. Furthermore, increased p15^INK4b^ mRNA transcription has been reported following EBNA3C inactivation in conditional systems, demonstrating EBNA3C involvement in p15^INK4b^ repression [13,58]. As with *CDKN2A,* ChIP analyses have indicated EBNA3A and EBNA3C bind near the transcription start sites of the *CDKN2B* locus, suggesting EBNA3A and EBNA3C play a direct repressive role [16,43,58]. In addition to p15^INK4b^ and p16^INK4a^, EBNA3A and EBNA3C have also been shown to repress the INK4 family member p18^INK4c^ through PRC2-mediated deposition of H3K27me3 ([13]).

### 4.2. EBNA3A- and EBNA3C-Mediated Transactivation of Oncogenic miRNAs That Target CDKIs

As well as directly repressing genes of the CDKI INK4 family, EBNA3A and EBNA3C can indirectly repress cell cycle components through transcriptional activation of repressive microRNA (miR). EBNA3A and EBNA3C were recently shown to mediate transactivation of the oncogenic miR cluster miR221 and miR222 (miR221/222), leading to repression of the CDKI p57^KIP2^, and to a lesser extent, p27^KIP1^ [12]. 

Screening LCLs, where EBNA3A and EBNA3C were either conditionally expressed or knocked out, identified modified expression of various miRNAs by the EBNA3 proteins. Expression of the oncogenic miR221/222 cluster was shown to be reduced when either EBNA3A or EBNA3C was non-functional, with a significant increase in miR221/222 mRNA when EBNA3A and EBNA3C were conditionally activated. ChIP analysis confirmed EBNA3A and EBNA3C binding sites within an enhancer element of the pri-miR221/222 locus, from which miR221 and miR222 are processed. ChIP analysis further revealed an altered epigenetic profile in which the transcriptional activation markers H3K4me3, H3K9ac, H3K27ac, and phospho-Ser5 Pol II were elevated at the transcription start site only when EBNA3A and EBNA3C were functionally expressed. Furthermore, chromosome confirmation capture established that long range chromatin interactions between EBNA3A and EBNA3C binding sites within the predicted enhancer site of pri-miR221/222 and its promoter only occur when EBNA3A and EBNA3C are functionally active. The miRNA221/222 cluster targets the CDKIs p57^KIP2^ and p27^KIP1^; transcriptional activation of the miRNAs by EBNA3A and EBNA3C leads to repression of p57^KIP2^, and to a lesser extent, p27^KIP1^. This provides a novel indirect method of cell cycle regulation mediated by EBNA3A and EBNA3C gene activation [12]. 

Alongside indirect repression of p27^KIP1^ and p57^KIP2^, EBNA3A and EBNA3C have also been associated with repression of the CDKI and CIP/KIP family member p21^WAF1/CIP1^ [59,60]. In contrast to the direct polycomb-mediated transcriptional repression of the INK4 proteins, or miRNA-mediated translational regulation of p57^KIP2^ and p27^KIP1^, EBNA3C works directly though interactions with the oncogenic kinase Pim-1 to result in proteasomal degradation of p21^WAF1/CIP1^ [59]. There is compelling evidence that transcriptional repression of p16^INK4a^ is the dominant factor in EBNA3A- and EBNA3C-mediated cell cycle control during B cell transformation by EBV [43]. However, EBNA3A and EBNA3C have been shown to target many CDKIs, employing diverse mechanisms that encompass regulation at the transcriptional, translational, and protein level (summarised in Figure 2). This suggests the EBNA3 proteins function to achieve a specific molecular balance that allows control of the cell cycle by EBV, enabling establishment of viral latency. 

## 5. Anti-Apoptotic Functions of EBNA3A and EBNA3C

Concurrent with manipulation of CDKI and cell cycle control, EBNA3A and EBNA3C further facilitate virally-induced proliferation and oncogenesis by repressing apoptotic responses to viral infection and transformation. Using a panel of EBNA3KO viruses in BL31 BL-derived cells, EBNA3A and EBNA3C were found to cooperatively repress the tumour suppressor and pro-apoptotic BCL-2-family member BIM (Bcl-2-interacting mediator of cell death) encoded by *BCL2L11* [9]. EBNA3-mediated regulation of BIM was also observed in LCLs, with repression shown to be reversible using an EBNA3C conditional system, whereby inactivating EBNA3C for three weeks led to elevated BIM mRNA levels [15,43]. BIM becomes upregulated during unscheduled cellular proliferation and directly initiates apoptosis through inhibition of BCL2 and other pro-survival proteins. This is particularly important during the development of lymphocytes, as BIM mediates negative selection of auto-reactive B cells, and initiates programmed cell death in B cells expressing low-affinity antibodies in the germinal centre [61,62,63]. Furthermore, BIM is activated by the protooncogene *MYC*, which is expressed by B cells following EBV infection and induces hyperproliferation. A hallmark of BL is translocation of *MYC* and subsequently constitutively elevated expression [64]. BIM is the effector of one of the accumulative apoptotic pathways triggered by MYC overexpression (the other being the p14^ARF^–MDM2–p53 pathway) and it has been shown that even loss of a single *BIM* allele accelerates B lymphomagenesis [65]. The epigenetic mode of BIM repression by EBNA3A and EBNA3C may be important for Burkitt’s lymphoma development, since it can create an environment permissive for MYC overexpression, even after EBNA3A and EBNA3C expression is shut down, as the virus progresses through different stages of latency [9,66,67].

Analogous with the epigenetic repression of INK4 family proteins, detailed ChIP analysis of EBV-positive B cells indicated that EBNA3-mediated repression of BIM involves modification of chromatin marks. This includes a reduction of histone acetylation and deposition of the H3K27me3 repressive mark within the *BCL2L11* locus, facilitating transcriptional repression [66]. Direct participation of EBNA3 proteins in repression of BIM was subsequently demonstrated when binding of EBNA3C to sites proximal to the *BCL2L11* transcriptional start site was confirmed by ChIP analysis, with later studies showing concurrent EBNA3A binding sites [15,42]. Furthermore, EBNA3A and EBNA3C were found to recruit the PRC2 subunits SUZ12 and EZH2 to BIM regulatory elements, facilitating H3K27me3 deposition [15]. A requirement for the polycomb system in EBV-mediated repression of BIM was further confirmed as BIM repression was found to be disrupted by lentiviral delivery of shRNA against PRC2 components, and reversed by an EZH2 inhibitor [15,42]. EBNA3A- and EBNA3C-mediated repression of BIM was also recently shown to involve long range chromatin interactions between the BIM promoter and enhancers bound by EBNA3A and/or EBNA3C [42]. 

In addition to EBNA3A- and EBNA3C-mediated repression of the important tumour suppressors p16^INK4a^ and BIM, alongside others mentioned in this article, EBNA3C has been shown to attenuate the DNA damage response (DDR), which functions to limit aberrant proliferation by inducing senescence and apoptosis [68]. The DDR is transiently activated in response to the hyperproliferation of EBV infected cells during primary infection. However, EBNA3C was shown to attenuate this response by inhibiting the DDR kinases CHK2 and ATM [68]. Induction of the DDR temporally correlates with the transient increase in p16^INK4a^ expression seen following primary infection [43]. This is associated with oncogene activation, but is also speculated to be a consequence of DNA damage due to hyperproliferating cells inappropriately entering the cell cycle S phase [43,53]. The continuation of the DDR, as well as p16^INK4a^ and BIM activation, is subsequently prevented by the expression of functional EBNA3C and EBNA3A, which help to prevent apoptosis and cell cycle arrest pathways and allow outgrowth of LCLs [43,53,68]. Furthermore, EBNA3C has also been associated with the repression of other factors involved in the cell cycle, DNA damage response, apoptotic pathways and the oncogenic stress response, including MDM2, p53, H2AX, pRb, E2F1, Cyclin A, ubiquitin ligase SCF^SKP2^ and c-Myc [69,70,71,72,73,74,75,76]. This well-documented oncogenic activity of both EBNA3A and EBNA3C is in stark contrast to the functions of the third EBNA3 protein, EBNA3B.

## 6. EBNA3B: The Antagonistic Tumour Suppressive EBNA3 Protein

Despite structural and partial sequence homology, EBNA3B has distinctly different functions from those of the oncogenic EBNA3A and EBNA3C. It is completely dispensable in vitro, and B cells infected with EBV in the absence of EBNA3B exhibit elevated proliferative capacity compared to WT [20,27,77]. This phenotype contrasts with that of the reduced efficiency of B cell transformation by EBNA3A-null EBV and the inability of EBNA3C-null EBV to cause LCL outgrowth [14,23,26,43].

In contrast to the oncogenic functions of EBNA3A and EBNA3C, EBNA3B appears to have tumour suppressive activity in vivo [27]. In NOD-scid-*IL2Rγ^null^* mice engrafted with human immune system components, which are susceptible to EBV infection, those infected with EBNA3B-KO exhibited increased splenomegaly and a higher frequency of large tumour masses than WT and EBNA3B-revertant infections after four weeks. These tumours destroyed splenic architecture, and by immunohistochemistry were monomorphic and highly proliferative, resembling the activated B cell (ABC) subtype of DLBCL. Furthermore, tumours derived from EBNA3B-deficient infections lacked infiltrating T cells. This was despite these infections priming a stronger systemic immune response compared to WT and revertant virus infections, with a higher percentage of splenic T cells, including CD8^+ve^ T cells capable of killing EBV-positive cells in vitro [27]. This is potentially a result of an absence of EBNA3B-mediated activation of the T cell migratory chemokine CXCL10, although microarray studies have identified other chemokine, chemokine receptor, and integrin genes whose expression is altered in EBNA3B-KO LCLs that could also contribute to the exclusion of T cells from the tumour [8,27]. Together, this indicates that EBNA3B plays a role in immunological trafficking and T cell surveillance of tumours. Furthermore, EBV-positive ABC-DLBCL with non-sense mutations in the EBNA3B gene, and BL and HL with in frame deletions, have also been identified in humans, suggesting that EBV mutations can contribute to occurrence of these cancers [27]. 

Despite the seemingly opposite basic functions of EBNA3B compared to EBNA3A and EBNA3C, EBNA3B cooperatively regulates some genes with EBNA3C, sometimes with the additional contribution of EBNA3A [8]. This is consistent with the extensive colocalisation of the EBNA3 proteins across the genome [18]. The EBNA3 proteins, working independently and in combination, seem to provide a complex balance during infection between the pro-proliferative functions of EBNA3A and EBNA3C, against the seemingly anticancer functions of EBNA3B. This compromise between the EBNA3 proteins has likely evolved to minimise the oncogenic risk to latent carriers while establishing persistence within the population.

## 7. Suppression of Plasma Cell Differentiation: An Additional Role for the Oncogenic EBNA3 Proteins

Aside from manipulating regulation of cell cycle factors, microarray analysis has also indicated that EBNA3 proteins may alter the regulation of various factors associated with B cell differentiation [8]. Recently, insight into this alternative EBNA3 function was demonstrated when EBNA3A and EBNA3C were shown to directly epigenetically repress the CDKI and plasma cell differentiation factor p18^INK4c^, encoded by *CDKN2C* [13,78,79,80], and BLIMP-1, the major transcriptional regulator of plasma cell differentiation and tumour suppressor, encoded by *PRDM1* [13,81,82,83].

Using a recombinant virus, in which EBNA3A and EBNA3C are both expressed conditionally, simultaneous inactivation of EBNA3A and EBNA3C during primary B cell infection was shown to lead to elevated levels of p18^INK4c^ and BLIMP-1 mRNA and protein [13]. In contrast to the INK4 proteins p15^INK4b^ and p16^INK4a^, functional expression of either EBNA3A or EBNA3C individually is largely sufficient to repress p18^INK4c^ and BLIMP-1 transcription [13,43,54,58]. The increased levels of p18^INK4c^ and BLIMP-1 in EBNA3A/EBNA3C-null cells were physiologically comparable to those of plasma cell models, and transcription of the BLIMP-1 target genes *XBP-1*, *LMO2*, and *SPIB* responded as expected to the elevated BLIMP-1 levels. Concurrent with the roles of p18^INK4c^ and BLIMP-1 in promoting plasma cell differentiation, flow cytometric analysis of EBNA3A/EBNA3C-null cells showed the development of a plasma cell-like phenotype, with elevated surface markers characteristic of plasma cells, including CD138 and CD38, alongside elevated IgG and IgM expression. This indicates that EBNA3A and EBNA3C have evolved, not only as oncogenic factors, but also as repressors of plasma cell differentiation [13]. This is likely a mechanism to promote transition of activated B-blasts through the germinal centre and subsequent differentiation of memory B cells, allowing establishment of the long-lived reservoirs of EBV within latently infected individuals [3]. 

As with the *BCL2L11, CDKN2A,* and *CDKN2B* loci, ChIP analysis showed EBNA3A and EBNA3C bind around the transcriptional start sites of the *CDKN2C* (p18^INK4c^) and *PRDM1* (BLIMP-1) genes, where they mediate deposition of H3K27me3 catalysed by the EZH2 component of the PRC2 complex. This leads to stable epigenetic reprogramming of these plasma cell differentiation associated genes [13]. In contrast to EBNA3-mediated repression of BIM, p15^INK4b^ and p16^INK4a^, which can be reversed by inactivation of either EBNA3A or EBNA3C, regulation of p18^INK4c^ and BLIMP-1 was shown to be irreversible by eight days post infection, with inactivation of EBNA3A and EBNA3C failing to lead to reactivation of either gene, even after thirty days [10,13,15,43,54]. The mechanisms responsible for the irreversible epigenetic reprogramming of p18^INK4c^ and BLIMP-1 by EBNA3 proteins are presently unknown, but may involve additional chromatin modifications. The differential regulation of these target genes highlights the complexity of EBNA3A and EBNA3C appropriation of the polycomb system.

EBNA3A- and EBNA3C-mediated repression of the plasma cell differentiation factors p18^INK4c^ and BLIMP-1 appears to be independent of EBNA3B [13]. However, microarray analysis of BL31 BL-derived cells suggests a distinct role for EBNA3B in B cell differentiation, as its deletion leads to upregulation of a subset of genes usually repressed in the germinal centre [8]. This suggests EBNA3B may contribute to the progression of EBV-infected cells through the germinal centre reaction, therefore helping facilitate memory B cell differentiation. While the role of EBNA3B in this capacity has not been investigated, it is possible that all of the EBNA3 proteins are directing differentiation towards the site of long-term latency. 

## 8. Summary

The complex cooperative and antagonistic functions of the EBNA3 proteins, encompassing the critical epigenetic reprogramming of target genes, are central to achieving EBV persistence and latency. EBV infection stimulates proliferation of infected B cells to facilitate differentiation. This aberrant cell proliferation is permitted due to EBNA3A- and EBNA3C-mediated repression of cell cycle arrest pathways and suppression of pro-apoptotic signals. As B cell differentiation may lead to plasma cells, EBV must also promote transition through the germinal centre to achieve latency in memory B cells. EBNA3A and EBNA3C thus also suppress plasmacytoid differentiation, supporting the creation of a long-lived reservoir of EBV-infected memory B cells. It is possible that this process is further supported by EBNA3B, through T cell interactions and additional regulation within the germinal centre. In addition, EBNA3B plays a role in suppressing the detrimental effects of EBNA3A- and EBNA3C-mediated oncogenic functions. Because the EBNA3s largely epigenetically reprogram genes, the regulation of those genes is sustained following the sequential silencing of viral gene expression during germinal centre transition. This ensures the memory B cell pathway is favoured during latency progression, but it is possible that long-term effects may contribute to the prevalence of EBV-associated cancers. The complex essential roles of EBNA3A, EBNA3B and EBNA3C within EBV infection thus appear to be delicately balanced, allowing this potent oncogenic virus to achieve ubiquitous and life-long latent infection in the worldwide population.
